# Cas13-based amplification-free quantification of aberrant RNAs during influenza virus infection

**DOI:** 10.1101/2023.11.03.565460

**Published:** 2023-11-03

**Authors:** Caitlin H. Lamb, Emmanuelle M. Pitré, Elizaveta Elshina, Charlotte V. Rigby, Karishma Bisht, Hamid Jalal, Cameron Myhrvold, Aartjan J.W. te Velthuis

**Affiliations:** 1 Department of Molecular Biology, Princeton University, Princeton, NJ 08544; 2 University of Cambridge, Department of Pathology, Addenbrooke’s Hospital, Cambridge CB2 2QQ, United Kingdom; 3 Public Health England, Addenbrooke’s Hospital, Cambridge CB2 2QQ, United Kingdom; 4 Department of Chemical and Biological Engineering, Princeton University, Princeton, NJ 08544; 5 Omenn-Darling Bioengineering Institute, Princeton University, Princeton, NJ 08544; 6 Department of Chemistry, Princeton University, Princeton, NJ 08544

**Keywords:** influenza virus, mini viral RNA, RNA polymerase, Cas13, CRISPR, innate immune, RIG-I

## Abstract

Influenza A virus RNA synthesis produces full-length and aberrant RNA molecules, which include defective viral genomes (DVG) and mini viral RNAs (mvRNA). Sequencing approaches have shown that several hundred unique aberrant RNA species may be present during infection, and that they can vary in size, segment origin, and sequence. Moreover, a subset of aberrant RNA molecules can bind and activate host pathogen receptor retinoic acid-inducible gene I (RIG-I), leading to innate immune signaling and the expression of type I and III interferons. Understanding the kinetics and distribution of these immunostimulatory aberrant RNA sequences is important for modeling the outcomes of IAV infection. We here first show that reverse transcription and PCR steps can yield imperfect aberrant RNA quantification data in a sequence-dependent manner. Next, we developed an amplification-free LbuCas13a-based detection method to quantify mvRNA amplification kinetics and subcellular distributions. We show that our assay can quantify the copy numbers of 10 specific mvRNA sequences in total RNA from cell culture, animal tissue or clinical nasopharyngeal swab extracts. In addition, we find kinetic and distribution differences between immunostimulatory and non-immunostimulatory mvRNAs, as well as mvRNAs derived from different segments, during infection. Overall, our results reveal a hitherto hidden diversity in the behavior of IAV mvRNAs and they suggest that their production is linked to replication of the individual viral segments. Cas13 is therefore a valuable new tool in our repertoire for investigating the impact of aberrant RNAs on RNA virus infection.

## Introduction

Influenza A viruses (IAV) cause mild to severe respiratory disease in humans. Upon infection, IAV releases eight segments of negative-sense, single-stranded RNA that are organized into viral nucleoprotein (vRNP) complexes. Each vRNP complex consists of a viral RNA (vRNA) that is bound by a helical coil of nucleoproteins (NP) and a copy of the RNA-dependent viral RNA polymerase (1, 2). During replication, the IAV RNA polymerase can produce a wide variety of aberrant RNA products including defective viral genomes (DVG) and mini viral RNAs (mvRNA), which contain the conserved termini of the viral genome segments but lack internal sequences (3–7). Some of these aberrant RNAs have been shown to bind and activate retinoic-acid inducible gene I (RIG-I), leading to downstream innate immune signaling (5, 8–11). The production of aberrant RNAs plays a key role in determining the pathogenicity of a viral infection and the timing of their production can affect morbidity and mortality in animal models (5, 10). In addition, the abundance of mvRNA levels was associated with the appearance of disease markers in mouse and ferret infections with highly pathogenic IAV strains (5, 10). However, not all aberrant RNA molecules are able to induce an innate immune response and in IAV, as well as other negative sense RNA viruses, sequence-specific preferences for RIG-I binding and activation have been observed (6, 9, 12, 13). For IAV, we recently showed that mvRNAs containing a template loop (t-loop), a transient RNA structure that can affect RNA polymerase processivity, are more potent inducers of the innate immune response than mvRNAs without a t-loop in cell culture (9), although the sensitivity to t-loops is IAV RNA polymerase-dependent (14). Given the large variety of aberrant RNA sequences produced during IAV infection and the specific effects of different sequences, it is becoming more important to develop methods to study the kinetics and impact of individual aberrant RNA sequences during infection.

**Figure 1.**
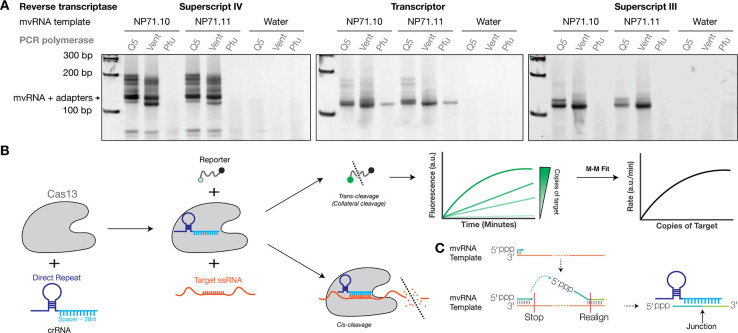
Detection of synthetic mvRNAs by different RT and PCR enzymes. **A**) Two synthetic mvRNAs were reverse transcribed into cDNA by three different RT enzymes and subsequently amplified by three different PCR enzymes. For sequences used, see Table S1. **B**) Schematic of RNA detection using Cas13. **C**) During IAV replication mvRNAs can be generated through a copy-choice mechanism that deletes internal genome segment sequences. crRNAs were designed to target the unique junction sequences, thereby distinguishing mvRNAs from full length viral genome segments and other mvRNAs.

Current tools to detect and quantify aberrant RNAs, such as mvRNAs, include primer extension, reverse transcription quantitative PCR (RT-qPCR), and next generation sequencing (15). To investigate if these methods can be used to correctly quantify different aberrant RNA species, we compared the ability of these methods to quantify known synthetic mvRNA sequences. We chose to perform this comparison on mvRNAs, because their short length (approx. 56–125 nt) facilitated chemical RNA synthesis and thus the possibility to use a well-defined input. Using two mvRNAs of the same length, but a different internal sequence, we observed a lack of consistency in the ability of three different RT and PCR enzyme combinations to produce a single amplification product (Fig. 1A). Additionally, the different RT and PCR enzyme combinations amplified the mvRNAs to different levels even though a fixed amount of the synthetic mvRNAs was provided as input (Fig. 1A, S1). Thus, while primer extension, PCR, and next generation sequencing are powerful methods to visualize or discover aberrant IAV RNAs, their use as quantification methods is limited by the enzymes used for the conversion or amplification of different mvRNA sequences.

To complement the above assays and perform mvRNA quantification without RT and amplification bias, we explored the use of Cas13, an RNA-guided nuclease, for the direct detection and quantification of mvRNAs (Fig. 1B). Cas13 uses a CRISPR RNA (crRNA) that consists of a direct repeat region, which is specific to the Cas13 ortholog, and a spacer region, which is designed to be complementary to the target RNA (16, 17). When CRISPR-Cas13 binds to the target RNA, the nuclease activity of Cas13 is activated. Cas13 can subsequently cleave the target, a process that is called *cis*-cleavage, as well as any nearby single-stranded RNA molecules, a process called trans-cleavage or collateral cleavage (18). The trans-activity can be measured with a reporter RNA molecule containing a fluorophore and a quencher (Fig. 1B), making Cas13 suitable for a wide-range of applications (19–24). Additionally, the measured fluorescent signal is proportional to the amount of target RNA present in a sample and the exact number of target RNA molecules present in a reaction can be calculated using a standard curve specific for the target RNA (Fig. 1B) (25). A downside of this approach is that a standard curve for each target must be used, as the Cas13 activity varies across target sequences.

Here, we developed a Cas13-based detection method to quantify the expression levels for 10 mvRNAs derived from five different IAV segments. We designed our crRNA such that it targeted the unique junction sequence formed upon mvRNA production (Fig. 1C). We tested our assays using total RNA from cell culture, animal tissue, or clinical nasopharyngeal swabs. By targeting mvRNAs specifically, we were able to use short, synthetic RNA standards to optimize and validate our assay. Moreover, due to the use of LbuCas13a, our assay is amplification-free and simple to use. Synthetic RNA standards were used to correct any sequence-specific variations in LbuCas13a activity and convert the fluorescent signal into mvRNA copy numbers. Using our assay, we observe kinetic and subcellular distribution differences between immunostimulatory and non-immunostimulatory mvRNAs, as well as mvRNAs derived from different segments. These results provide new insight into the diversity and behavior of aberrant RNAs during IAV infection in cell culture, ferrets, and humans.

## Results

### LbuCas13a can be used to detect and quantify mvRNAs without amplification.

**Figure 2.**
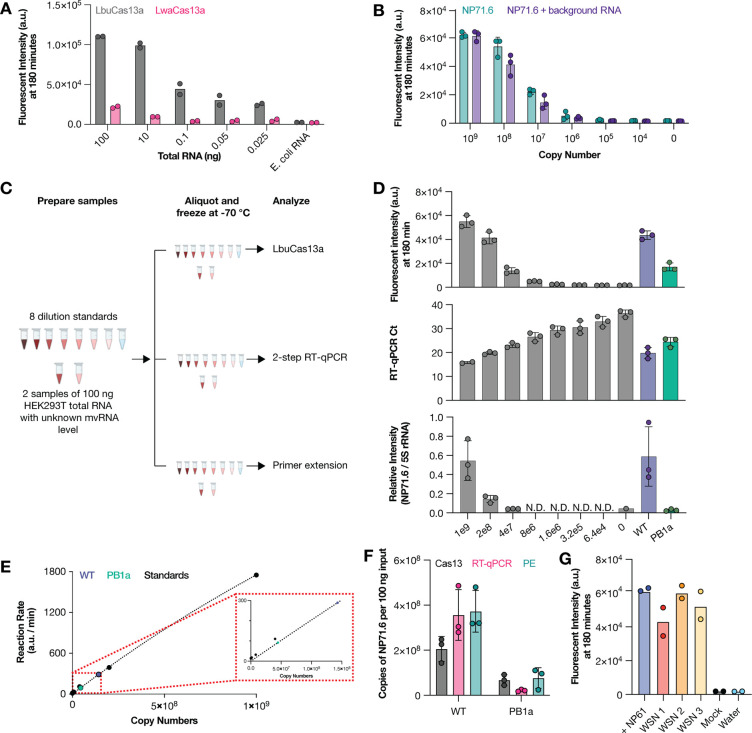
Detection of mvRNAs using LbuCas13a. **A**) Detection of 5S rRNA using LbuCas13a or LwaCas13a. Data points indicate two biological repeats. **B**) Detection synthetic mvRNA NP71.6 diluted in water or HEK293T total RNA using LbuCas13a. Data points indicate three independent repeats. **C**) Schematic of RNA sample preparation for mvRNA detection. **D**) Detection of synthetic or transfected mvRNA NP71.6 using LbuCas13a (top), TaqMan-based RT-qPCR (middle) or primer extension (bottom). Data points indicate three independent repeats. For raw primer extension data see Fig. S2. **E**) Michaelis-Menten fits to the maximum rate of fluorescence as a function of synthetic mvRNA NP71.6 copy number. **F**) Copy number of mvRNA NP71.6 in transfection samples. Data points indicate three biological repeats. **G**) Copy number of synthetic mvRNA NP-61 or mvRNA NP-61 in WSN infections of A549 cells. Data points indicate two technical repeats.

Cas13 orthologs have variable amounts of *trans*-cleavage activity (18). To determine if Cas13 could be used to detect short RNAs without the previously described amplification and T7 transcription steps, we compared the ability of two Cas13 orthologs, LbuCas13a and LwaCas13a, to detect human 5S rRNA in a dilution series of total RNA extracted from HEK293T cells. We observed that LbuCas13a was able to detect 5S rRNA with as little as 0.025 ng of total RNA input, whereas LwaCas13 only produced a signal above background when incubated with 10 ng of total RNA input (Fig. 2A).

We next investigated whether LbuCas13a could detect a previously described control mvRNA based on segment 5 (NP71.6). To detect NP71.6, we designed a crRNA with a spacer sequence complementary to the unique junction sequence that was created through deletion of internal IAV segment 5 sequences. We subsequently diluted in vitro synthesized NP71.6 in water or 100 ng of total HEK293T RNA and found that we were able to detect the mvRNA with a limit of detection <10^6^ copies, irrespective of the background used (Fig. 2B). No fluorescent signal was observed in the absence of synthetic NP71.6 (Fig. 2B). We subsequently explored whether we could use LbuCas13a to quantify unknown amounts of NP71.6 in total RNA extracted from HEK293T cells transfected with plasmids expressing the three IAV RNA polymerase subunits and the NP71.6 mvRNA. To this end, we first made a 5-fold dilution series of synthetic NP71.6 to create a standard curve (Fig. 2C; S2). Next, we calculated the maximum slope of the fluorescent Cas13 signal for each point of the standard curve, plotted the maximum slope against the mvRNA concentration, and fitted the resulting distribution to the Michaelis-Menten equation (Fig. 2D). These fitted standard curve data allowed us to convert the LbuCas13a fluorescent signal obtained for the plasmid-expressed mvRNA into mvRNA copy numbers present in the transfected cells (Fig. 2D, E). When we compared the quantification obtained using LbuCas13a to the results obtained using primer extension or a two-step Taq-man based RT-qPCR, we observed that our CRISPR-based detection method yielded no significantly different results (Fig. 2D, E). A similar result was obtained when we expressed the NP71.6 mvRNA in the presence of an inactive IAV RNA polymerase to measure RNA polymerase I (PolI)-derived input levels (Fig. 2E).

We subsequently investigated whether LbuCas13a was able to detect a specific mvRNA sequence during IAV infection. To increase the likelihood of detection, we designed a crRNA complementary to a 61 nt-long, highly abundant mvRNA derived from segment 5 (NP-61), which we had previously identified in ferret lung tissues infected with A/Brevig Mission/1/1918 (H1N1) (abbreviated as BM18) and A549 cells infected with A/WSN/1933 (H1N1) (abbreviated as WSN) (5, 9). As shown in Fig. 2F, LbuCas13a successfully detected synthetic NP-61 as well as NP-61 expressed during WSN infection of A549 cells in total RNA. No signal was detected in our water and mock infection controls (Fig. 2F). Overall, these results indicate that LbuCas13 can detect a specific mvRNA sequence in well-defined samples similar to primer extension and RT-qPCR, and that it can detect a specific mvRNA sequence in a complex RNA sample without sequence-specific biases that are potentially associated with RT and PCR steps, as shown in Fig. 1 and S1.

### mvRNAs can be detected from 3 hours post infection and accumulate over time.

**Figure 3.**
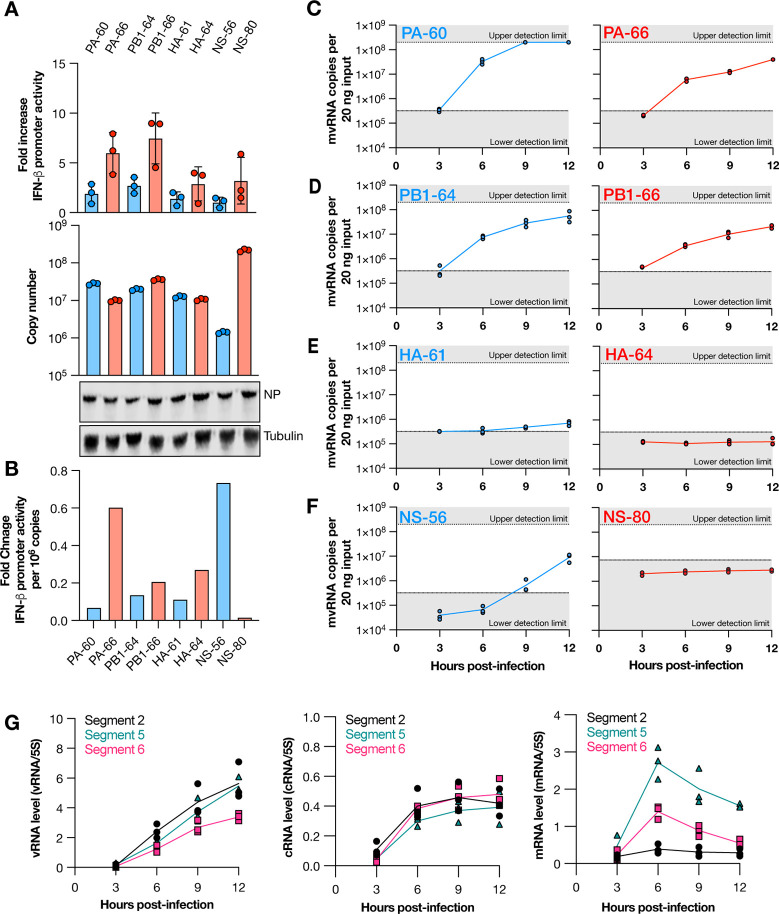
mvRNAs have different effects on IFN-β promoter activity and different kinetics during infection. **A**) IFN-β promoter activation (top graph) and copy number (bottom graph) of transfected mvRNA templates in the presence of the IAV RNA polymerase and NP. Western blot of NP expressed and tubulin loading control in shown in bottom images. **B**) IFN-β promoter activation per million mvRNA copies. **C-F**) Copy number of mvRNAs during WSN infection of A549 cells. **G**) Level of replication and transcription products during WSN infection of A549 cells as determined by primer extension. For denaturing PAGE analysis results see Fig. S3.

We previously identified many hundreds of different mvRNA sequences using next generation sequencing (5, 9). We therefore next sought to determine whether we could quantify different mvRNAs produced over the course of IAV infections and study their kinetics. However, while our LbuCas13-based assay does not require amplification, it is not high-throughput and depends on relatively costly synthetic RNAs to generate standard curves. We therefore focused on four sets of two mvRNA sequences derived from IAV segment 2, 3, 4 and 8 (encoding viral proteins PB1, PA, HA, and NS), covering the spectrum of genome segment sizes, segment-specific kinetic differences (26), and segment-specific unique mvRNA counts (9). We cloned these eight mvRNAs into RNA polymerase I-based (pPolI) expression plasmids and tested their ability to activate innate immune signaling by transfecting the plasmids in HEK293T cells alongside constructs expressing the IAV RNA polymerase subunits, a *Renilla* transfection control, and a luciferase based IFNβ reporter. Measurement of the luciferase signal showed that two mvRNAs, derived from segment 1 and 2 (PB1–66 and PA-66, respectively) were strong inducers of IFNβ promoter activity, while the other six mvRNAs were relatively weak inducers (i.e., PB1–64, PA-60, HA-61, HA-64, NS-56 and NS-80) (Fig. 3A).

We next designed crRNAs specific for the junction in each mvRNA (Fig. 1C) and used these crRNAs to quantify the number of mvRNA copies present using a synthetic RNA standard curve for each of the mvRNAs (Fig. 3A). Using the luciferase reporter data and the number of mvRNA copies identified, we were able to calculate the potential of each mvRNA to trigger an innate immune response (Fig. 3B). We observed that per segment, one mvRNA had a strong potential to induce IFNβ promoter activity, while the other mvRNA had a weak potential (Fig. 3B). With one exception (the NS segment), mvRNAs that had a higher copy number tended to have a lower potential to activate the innate immune response (Fig. 3B), which is generally in line with our previous observation on t-loop containing mvRNAs (9). The presence of a clear exception on the t-loop model suggests that at least one other, still undiscovered, mechanism must exist through which mvRNAs can activate the innate immune signaling.

Following the transfection-based characterization of the different mvRNAs and confirming our ability to detect them without amplification, we next measured the kinetics of the eight mvRNAs over a synchronized, 12-hour infection of A549 cells with WSN (Fig. 3C–F). We observed that mvRNAs PA-60, PA-66, PB1–64, PB1–66 were detectable in A549 cells as early as 3 hours post infection (hpi) and that their copy number increased steadily over time (Fig. 3 C,D). At 9 hpi, the PA-60 signal reached saturation, whereas the PA-66, PB1–64, PB1–66 signals did not reach this level in three biological repeats (Fig. 3D). In contrast, mvRNAs HA-61 and NS-56 were undetectable until 9 hpi (Fig. 3F) and both HA-64 and NS-80 remained undetectable over the course of the 12-hour infection (Fig. 3 E, F). In addition, we observed that three of the mvRNAs with a lower potential to activate the innate immune response (PA-60, PB1–64, and HA-61) were more abundant compared to mvRNAs of the same segment that had a stronger potential to activate the innate immune response (PA-66, PB1–66, and HA-64). Comparing the accumulation kinetics of the PB1 mvRNAs to the full-length segment 2, 5 and 6 kinetics, we found that the mvRNA accumulation followed the trend of the full-length vRNA segments, and not the mRNA (Fig. 3G, S3).

### PA-60 and PA-66 have different distributions in infected cells.

**Figure 4.**
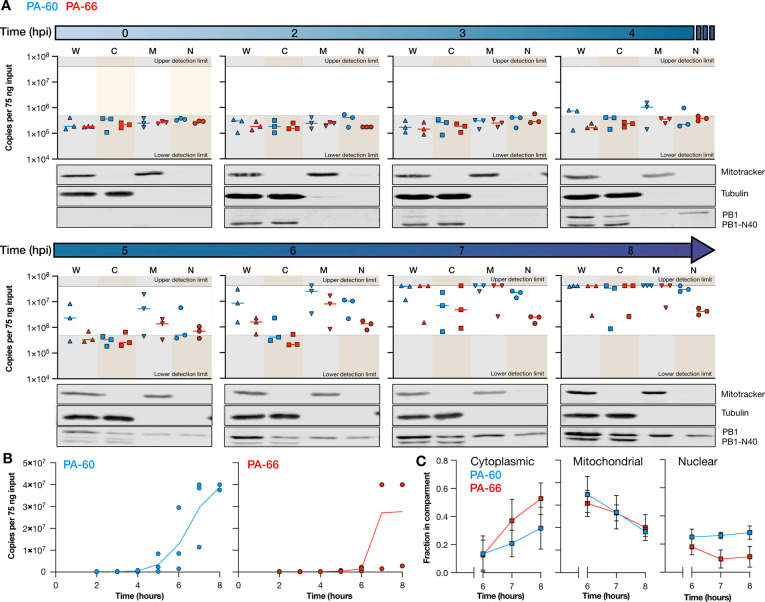
mvRNA PA-60 and PA-66 distribution in subcellular compartments. **A**) Copy number of mvRNAs PA-60 and PA-66 per 75 ng input RNA per subcellular compartment during WSN infection of A549 cells. Data points indicate three biological repeats. Western blots show fractionation of one biological repeat. For additional western blot data see Fig. S4. **B**) Total copy number of mvRNAs PA-60 and PA-66 during WSN infection of A549 cells per 75 ng of total RNA. Data points indicate three biological repeats. **C**) Fraction of mvRNAs PA-60 and PA-66 per subcellular compartment. Data points indicate an average of three biological repeats. Error bars indicate SEM.

We next sought to compare the subcellular distribution of the PA-60 and PA-66 mvRNA pair over the course of an 8-hour IAV infection. To this end, we performed synchronized infections of A549 cells with WSN and fractionated cells at 2, 3, 4, 5, 6, 7, and 8 hpi (Fig. 4A). During the fractionation process, the volumes are maintained constant so that protein samples can be compared directly to each other. However, in constant volumes, different fractions have different RNA levels. To ensure that the LbuCas13a-based reactions for the standard curve (diluted in HEK293T RNA) and samples were performed in a similar RNA environment, we normalized the RNA input for each fraction to 75 ng.

We first analyzed the total mvRNA levels in each fraction. As shown in Fig. 4A and B, we were not able to detect mvRNAs before 4 hpi in the whole cell extract, similar to the infection time course described in Fig. 3. From 4 hpi, the PA-60 and PA-66 levels started to increase, with PA-60 showing faster kinetics than PA-66. The production of PA-60 and PA-66 followed the detection of PB1 in the nucleus 3 hpi. At 7 hpi, both mvRNAs started to reach the upper limit of our detection assay in most biological repeats (Fig. 4A,B).

In the subcellular fractions (Fig. 4A), we found that PA-60 became first detectable in the nuclear and mitochondrial fractions at 4 hpi. The PA-60 cytoplasmic mvRNA levels remained undetectable until 6 hpi and then rose quickly. At 6 and 7 hpi, the PA-60 nuclear and mitochondrial levels started to the upper detection limit of our assay. The PA-66 mvRNA was slower to accumulate and became first detectable in the nuclear and mitochondrial fractions 5 hpi. At 7 hpi, the PA-66 mvRNA levels became measurable in the cytoplasm and then rose, reaching the upper limit of detection 7 hpi. The PA-66 mitochondrial levels also increased quickly, reaching the upper limit of detection at the same time as the PA-66 cytoplasmic levels. In contrast, the PA-66 nuclear levels deviated from this trend and accumulated only marginally during the course of the infection (Fig. 4A).

To compare the different fractions directly to each other, we corrected the measured LbuCas13a signal for the RNA input adjustment (Fig. S4) and plotted the distribution of the two mvRNAs as a fraction of the total (Fig. 4C). Interestingly, both PA-60 and PA-66 were initially enriched in the mitochondrial fraction. This enrichment then declined for both mvRNAs over the course of the infection. At 8 hpi, PA-60 reached an equal distribution among the three subcellular compartments. However, PA-66 became more enriched in the cytoplasm and only ~10% of the total signal was found in the nucleus at 8 hpi. This enrichment of PA-66 in the cytoplasm may contribute to the slower accumulation of PA-66 overall, as less PA-66 would be associated with active replication complexes. In addition, the enrichment of PA-66 in the cytoplasm increased its likelihood for detection by cytoplasmic pathogen receptors, such as RIG-I, and thereby its potential for activating the immune response. The enrichment of an immunostimulatory mvRNA in the cytoplasm is also in line with previous data showing that mvRNA templates that activate innate immune signaling become differentially enriched in the cytoplasm over the nucleus (10).

### LbuCas13a can detect mvRNAs in RNA extracted from ferret lungs and clinical swabs.

**Figure 5.**
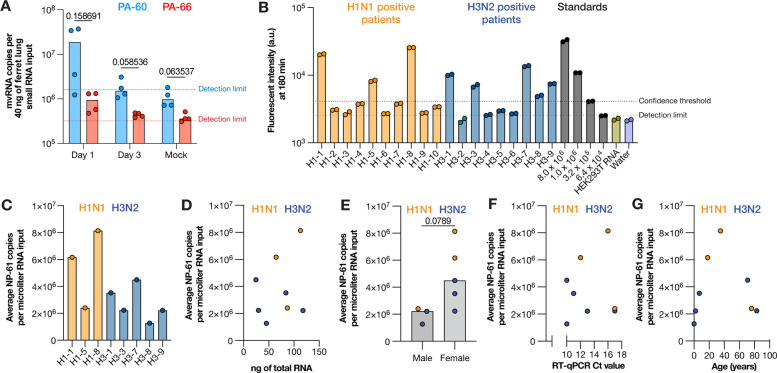
Detection of mvRNAs in ferret lung and clinical nasopharyngeal samples using LbuCas13a. **A**) Detection of mvRNAs PA-60 and PA-66 in small RNA (<200 nt) fractions extracted from ferret lung homogenates infected with BM18. **B**) Detection of mvRNA NP-61 in equal volumes of total RNA extracted from clinical nasopharyngeal samples. **C**) Copy number of mvRNA NP-61 in equal volumes of total RNA extracted from clinical nasopharyngeal samples for the samples that passed the confidence threshold. **D**) Copy number of mvRNA NP-61 plotted against the amount of ng total RNA used, **E**) plotted against the patient sex, **F**) plotted against clinical RT-qPCR Ct value, **G**) plotted against patient age. P-values indicated are based on non-parametric t-test.

We had previously shown that mvRNAs can be detected in mouse and ferret lung samples using RT-PCR and next generation sequencing. Following the detection and characterization of several mvRNAs using LbuCas13a in cell culture, we next sought to determine whether we could use the same approach to detect and quantify the PA-60 and PA-66 mvRNAs in RNA extracted from ferret lung samples infected with BM18. As shown in Fig. 5A, both PA-60 and PA-66 were detectable on day 1 and day 3 post infection, and not in the mock infected ferret samples, apart from one PA-66 reaction. In line with our tissue culture analyses (Fig. 3, 4), we found that the PA-60 copy number was on average higher than the PA-66 mvRNA level (Fig. 5A).

We next determined whether we could detect mvRNAs in RNA extracted from IAV positive clinical nasopharyngeal swabs. The clinical samples obtained were either positive for seasonal H1N1 or H3N2, and not co-infected with other respiratory pathogens, as determined by clinical RT-qPCR. We first confirmed that we could detect mvRNAs in clinical samples using a non-quantitative RT-PCR and found signals corresponding to the expected size in 6 of 10 IAV positive clinical samples (Fig. S5). Next, we tested our LbuCas13a assay, but failed to detect PA-60 or PA-66 in the clinical samples. As PA-60 and PA-66 have relatively low abundance based on our previous sequencing data, we subsequently attempted to detect the highly abundant mvRNA NP-61 that we can detect in tissue culture samples (Fig. 2G). As shown in Fig. 5B, we observed positive signals above our limit of detection for 18 of the 19 clinical samples. However, out of an abundance of caution, we set our confidence threshold at the lowest dilution of our standard curve with synthetic NP-61. Using this more stringent threshold, we were able to detect NP-61 in 8 out of 19 IAV positive clinical samples (Fig. 5B). We next used our synthetic NP-61 mvRNA standard curve to calculate the number of NP-61 copies per μl of extracted clinical sample (Fig 5C). Further analysis showed that the NP-61 copy numbers were not correlated with the amount of RNA input (Fig. 5D), or the reported clinical RT-qPCR Ct value (Fig. 5E), patient sex (Fig. 5F), or patient age (Fig. 5G). Overall, these findings show that mvRNAs can be detected and quantified in a wide-range of samples, including influenza virus positive samples. We expect that future research can now address when mvRNAs first appear in clinical samples and if their production contributes to the activation of the innate immune response in humans.

## Discussion

During IAV infection, aberrant RNA molecules of various lengths and sequences are produced. A subset of these molecules can activate the innate immune response, making them important targets for in-depth characterization. We observed that routinely used RNA quantification methods were sensitive to the mvRNA sequence and we therefore explored the RNA-guided enzyme Cas13 as an additional tool to quantify mvRNAs levels in cell culture, animal model, and patient samples. We first showed that the LbuCas13a ortholog can detect and quantify mvRNAs with a higher sensitivity than primer extension (Fig. 2), and that LbuCas13a reaction can avoid errors or biases introduced by RT-PCR-based amplification (Fig. 1A), because it can detect mvRNAs in an amplification-free manner. In a sample containing an mvRNA expressed in cells and amplified by the IAV RNA polymerase, LbuCas13 quantifications yielded similar results to RT-qPCR and primer extension, although minor differences were observed (Fig. 2). On the one hand, the differences in mvRNA copy number between the Cas13-based quantification and the primer extension and RT-qPCR quantification may derive from additional RT or RT-PCR products created by the conversion and amplification enzymes (Fig. 1). On the other hand, the IAV RNA polymerase can produce additional aberrant products from an input mvRNA in cell culture. These products would be converted to cDNA by an RT and amplified by PCR as they (could) contain the same primer binding sites. However, the Cas13 enzyme would only detect them if they contained the junction site to which the crRNA can bind. This problem could potentially be minimized in TaqMan-based RT-qPCRs by using a TaqMan probe that hybridizes over the unique junction. Finally, different assays have different signal-to-noise ratios. This can be a particular issue in primer extension experiments when detecting low RNA levels. While our assay also has an advantage of RT or RT-qPCR assays by limiting the amplification bias, the lack of amplification does make our assay less sensitive than RT-qPCR.

We further showed that LbuCas13a can be used to quantify mvRNAs derived from five different IAV segments in transfected cells (Fig. 2 and 3), WSN-infected cells (Fig. 3 and 4), BM18 infected ferret lungs (Fig. 5), and seasonal H1N1 and H3N2 infected clinical samples (Fig. 5). In the tissue culture experiments, we observed that mvRNA dynamics vary among different mvRNA sequences and that they accumulate over the course of infection, similar to the full-length vRNA segments. In agreement with previously published data, we also showed that mvRNAs with a potential to induce a stronger innate immune response are less abundant than mvRNAs that induce a weaker innate immune response for the four segments we examined (Fig. 3). Our data also reproduce the finding that mvRNA abundance does not necessarily correlate with innate immune activation, but they also show that the two mvRNAs from the NS segment do not follow this model and that other mechanisms of innate immune activation by IAV mvRNAs remain to be discovered (Fig. 3). Interestingly, two of the cloned mvRNAs, which had originally been identified through RNA sequencing, were not detected in A549 cell infections. Presently, it is unclear if these mvRNAs are of exceptionally low abundance and stay below the detection limit, or if these sequences were artifacts of the RT-PCR steps used to make the sequencing library. To address this question and obtain a better estimate of the misidentified mvRNA sequences in the future, a high-throughput, multiplexed version of our assay must be developed and combined with a systematic analysis of all identified (and potentially unknown) mvRNA sequences. These observations are likely also important for studies of DVG sequences, which also rely on RT-PCR-based sequencing libraries to look at sequence variation.

We also examined one immunostimulatory and non-stimulatory mvRNAs derived from segment 3 (PA-66 and PA-60) and investigated the subcellular distribution and accumulation of these mvRNAs during infection. We observed that while both stimulatory and non-stimulatory mvRNAs appear in mitochondria early in infection, the immunostimulatory mvRNA then accumulates in the cytoplasm whereas the non-stimulatory mvRNA becomes evenly distributed over the nuclear, cytoplasmic, and mitochondrial fractions (Fig 4B, C). Presently, the mechanism underlying these different distributions is unknown, but the observation agrees with our study of transfected mvRNAs and may help explain why the immunostimulatory mvRNA is able to activate the innate immune response better. Given the early presence of both mvRNAs in the mitochondrial fraction, it is surprising that we do not observe a strong innate immune response in the presence of both mvRNAs. It is possible that low mvRNA levels are insufficient to activate the innate immune response and that higher mvRNA levels are needed to overcome the negative feedback loops that prevent RIG-I from activating the MAVS-dependent signaling cascade (27).

Finally, we showed that the difference in abundance between the segment 3 immunostimulatory and non-stimulatory mvRNAs is also present in ferret lungs one day after infection with BM18 (Fig. 5A). In addition, we were able to measure the copy number of a highly abundant mvRNA in clinical samples that were positive for H1N1 and H3N2 seasonal IAV RNA, demonstrating that our assay works for many different IAV strains and for many different sample types (Fig. 5B, C). However, despite detection of this mvRNA in these clinical samples, we are not able to establish any correlations between mvRNA copy number and clinical Ct value, amount of input material, patient age or patient sex (Fig. 5). We expect that any correlations were confounded by unknown clinical factors, as well as when patients were tested in the course of their infection, their immune status, sampling differences, and environmental factors. Future studies may be able to address the importance of these factors in detail.

In summary, understanding the kinetics and dynamics of IAV aberrant RNAs is important because various lines of research have shown that these RNAs can play a role in the outcome of infection. Here we used Cas13 to quantify and characterize 10 different IAV mvRNAs in detail and identify differences in their kinetics and distribution. While our assay is limited in scope due to its limited multiplexing capabilities, it reveals a fascinating, hidden diversity among IAV aberrant RNA molecules, and we hope that it will stimulate both the development of multiplexed approaches to study this as well as a more in-depth characterization of these molecules in the future in order to better understand their role in the outcome of disease.

## Materials and Methods

### Preparation of synthetic RNA and primers

Primers were synthesized by Integrated DNA Technologies (IDT) and resuspended in nuclease-free water to 100 μΜ. Primers were stored at −20 °C and were further diluted prior to analysis. crRNAs were synthesized by IDT and resuspended in nuclease-free water to 100 μM. crRNAs were stored at −70 °C and were further diluted prior to analysis. Synthetic RNA targets were synthesized by IDT and resuspended in nuclease-free water to 100 μM. Synthetic RNA targets were stored at −70 °C and were further diluted prior to analysis.

### Plasmids

The firefly luciferase reporter plasmid under the control of the *IFNβ* promoter [pIFΔ(−116)lucter] and the transfection control plasmids constitutively expressing *Renilla* luciferase under control of the thymidine kinase promoter (pTK-*Renilla*) were described previously (5, 9). The pcDNA3-based WSN protein expression plasmids and pPolI-based WSN template RNA expression plasmids were described previously (5, 9). The mvRNA expressed plasmids were generated by site-directed mutagenesis using the primers listed in Table S3.

### Cells

HEK293T and A549 cells were originally sourced from the American Type Culture Collection (ATCC). All cells were routinely screened for mycoplasma, and grown in Dulbecco’s modified Eagle medium (DMEM) containing pyruvate, high glucose, and L-glutamine (GeneDepot) with 10% fetal bovine serum (Gibco) at 37 °C and 5% CO_2_.

### Transfections

Transfections of HEK293T cells were performed using Lipofectamine 2000 (Invitrogen) and Opti-MEM (Invitrogen) as described previously (5, 9).

### Cell Culture Infections

For infections, 10 cm dishes were seeded with 5 × 10^6^ A549 cells 15 h before infection. Cells were infected with influenza A/WSN/33 at MOI 3. The cells were incubated with virus inoculum at 4°C for 1 h in DMEM/ 0.5% FBS to ensure synchronization of the infections. Next, the inoculum was removed, and the cells incubated at 37 °C in DMEM/ 0.5% FBS for 2, 3, 4, 5, 6, 7, 8 h. Mock-infected A549 cells were incubated for 8 h. At each time point, cells were fractionated using cell Fractionation Kit (Abcam) and RNA extracted using TRI Reagent (Molecular Research Center, Inc).

### RNA Sample preparation

RNAs extraction was carried out using Tri Reagent (Molecular Research Center, Inc) following the manufacturer’s instructions and as described previously (15). The RNA concentration was determined using a NanoDrop One spectrophotometer (Thermo Fisher) and diluted in RNase free water prior to analysis.

### RT-qPCR and primer extension

Primers were pre-annealed to the RNA by heating for 2 minutes at 95 °C prior to an RT step carried out using SuperScript III Reverse Transcriptase (Invitrogen) as described previously (15). After the RT step, probe-based qPCR was carried out using Luna Universal Probe qPCR Master Mix (New England Biolabs) and the QuantStudio3 Real-Time PCR System (Thermo Fisher) using manufacturer’s instructions. The primers and probes used are listed in Table S3. Primer extensions were performed and analyzed as described previously (5, 9).

### Cas13-based detection reactions

Each reaction contained 10nM LbuCas13a, 4 mM HEPES pH 8.0, 12 mM KCl and 1% PEG, 2U/μL RNAse inhibitor murine (New England Biolabs), 0.25 μM 6UFAM (Table S3), 14mM MgOAc, 5nM crRNA (Table S3), and the reported amount of target RNA. Each reaction was first combined in a volume of 44 μL in 96-well plates. After mixing, 20 μL was transferred in duplicate to 384-well plates. The plate was then placed in a BioTek Cytation 5 Cell Imaging Multi-Mode Reader (Agilent) and incubated at 37 °C for 3 hours. Fluorescence was measured every 5 minutes.

### Curve fitting and Quantification

The maximum slope of each sample and standard was obtained by first determining the slope of every 3 data points starting at t = 0 min (0–10 min, 5–15 min …170 – 180 min). The maximum slopes of the standards were then plotted against the known copy numbers of the standards. Standards that reached saturation or quickly reached saturation (slopehigh-concentration << slopelow-concentration) were omitted prior to fitting the curve. The curve was fit to the Michaelis-Menten equation and K_m_ and V_max_ values were estimated using the Python 3 and the curve-fit trust region reflective (TRF) method from the scipy.optimize package. The V_max_ and K_m_ values were also obtained using Prism 10 software, both Python and Pris m 10 software produced the same V_max_ and K_m_ values. Using the obtained K_m_ and V_max_ values and the maximum velocity (V), referred to as the reaction rate in the text, of the sample, the unknown concentrations of the samples were estimated.

### Western blotting and antibodies

IAV proteins were detected using polyclonal anti-IAV PB1 protein antibody (GTX125923, GeneTex) and anti-IAV NP protein antibody (GTX125989, GeneTex), diluted 1:2000 in phosphate buffered saline (PBS)/5% BSA (RPI). To confirm fractionation of the A549 cells, Mitotracker (AB92824, Abcam), γ-tubulin (MCA77G, Bio-rad) and Histone H3 (AB1791, Abcam) antibodies were used, diluted 1:1000–5000 in PBS/5% BSA (RPI). Secondary antibodies IRDye 800 goat anti-rabbit (926–32211, LI-COR), and IRDye 680 goat anti-rat (926–68076, LI-COR) diluted 1:10,000 in PBS/5% BSA were used. Western blots were developed using the Odyssey CLx imaging system (LI-COR).

### Luciferase-based IFN-β promoter activation assays

Transfections to perform RNP reconstitutions and IFN-β reporter assays in HEK293T cells were essentially performed as described previously (5, 9). RNP reconstitutions were carried out in a 6-well plate format with cells (3×10^6^) seeded 24 hours prior to transfection. Per transfection, 250 ng of the pcDNA or pPPI4 plasmids encoding PB1, PB2, PA, NP and 250 ng of pPolI plasmid encoding a mvRNA were transfected alongside 100 ng of plasmid expressing firefly luciferase from the IFN-beta promoter and 10 ng of plasmid expressing *Renilla* luciferase using Lipofectamine 2000 (Invitrogen) to the manufacturer’s specifications. Twenty-four hpi, the medium was aspirated, and cells washed with 1 mL of PBS. Cells were resuspended in 200 μl PBS, of which 50 μl was used for the IFN-β promoter activity assay and the remaining cells were used for the Cas13 assay and western analysis. The IFN-β promoter activity assay was done in duplicate using 25 μl of cell suspension in PBS per well in a white 96-well plate format. Next, 25 μl of DualGlo reagent (Promega) was added per well, samples were incubated at RT for 10 min (in dark), and the firefly luciferase readings were taken using a Synergy LX Multimode Microplate Reader (Biotek). Twenty-five μl of Stop-Glo reagent/well was added next, plate was incubated for 10 min at RT (in dark), and the *Renilla* luciferase readings were taken. Firefly luciferase values were normalized by the *Renilla* luciferase values.

### Clinical samples

Nasopharyngeal samples were taken during routine testing from patients hospitalized at Addenbrookes Hospital during the 2016 – 2017, 2017 – 2018, 2018 – 2019 and 2019 – 2020 flu seasons. Patients were positive for either H1N1 or H3N2, but not other respiratory viruses, and samples were taken from a range of pathologies (asymptomatic to death). The study protocol was reviewed and approved by the Health Research Authority (IRAS ID 258438; REC reference 19/EE/0049). Per sample (typically 1.5 mL), 250 μl was used for total RNA extraction using Tri Reagent. RNA was dissolved in 10 μl RNase free water and stored at −70 °C prior to Cas13 analysis.

### Ferret lung samples

Ferret lung samples were kindly provided by Dr. Emmie de Wit. Ferret experiments were described previously (28) and leftover samples were inactivated and shipped according to standard operating procedures for the removal of specimens from high containment and approved by the Institutional Biosafety Committee. The original experiments were approved by Institutional Animal Care and Use Committee of Rocky Mountain Laboratories, National Institutes of Health, and conducted in an Association for Assessment and Accreditation of Laboratory Animal Care international-accredited facility according to the guidelines and basic principles in the United States Public Health Service Policy on Humane Care and Use of Laboratory Animals, and the Guide for the Care and Use of Laboratory Animals.

### Statistical Testing

GraphPad Prism10 software was used for statistical testing. Unless otherwise stated, error bars represent standard deviation and individual data points indicate biological repeats.

## Supplementary Material

Supplement 1
