# Supplementary material for: Quantification of influenza virus mini viral RNA dynamics using Cas13
Source: bioRxiv. 2024 May 2:2023.11.03.565460. Originally published 2023 Nov 3. Preprint. [Version 2] doi: 10.1101/2023.11.03.565460 (PMC10635118; doi:10.1101/2023.11.03.565460)
Supplement: Supplement 1 [file media-1.pdf]

Supplemental information for:

Quantification of influenza virus mini viral RNA dynamics using Cas13

Caitlin H. Lamb[1], Emmanuelle M. Pitré[1,2], Elizaveta Elshina[1], Charlotte V. Rigby[1,2,3], Karishma Bisht[1], Michael S. Oade[1], Hamid Jalal[3], Cameron Myhrvold[1,4,5,6,*], Aartjan J.W. te Velthuis[1,*]

[1] Department of Molecular Biology, Princeton University, Princeton, NJ 08544

[2] University of Cambridge, Department of Pathology, Addenbrooke's Hospital, Cambridge CB2 2QQ, United Kingdom

[3] Public Health England, Addenbrooke's Hospital, Cambridge CB2 2QQ, United Kingdom

[4] Department of Chemical and Biological Engineering, Princeton University, Princeton, NJ 08544

[5] Omenn-Darling Bioengineering Institute, Princeton University, Princeton, NJ 08544

[6] Department of Chemistry, Princeton University, Princeton, NJ 08544

[*]Address correspondence to: A.J.W.t.V. (aj.te.velthuis@princeton.edu); C.M. (cmyhrvol@princeton.edu)

Supplementary Figures

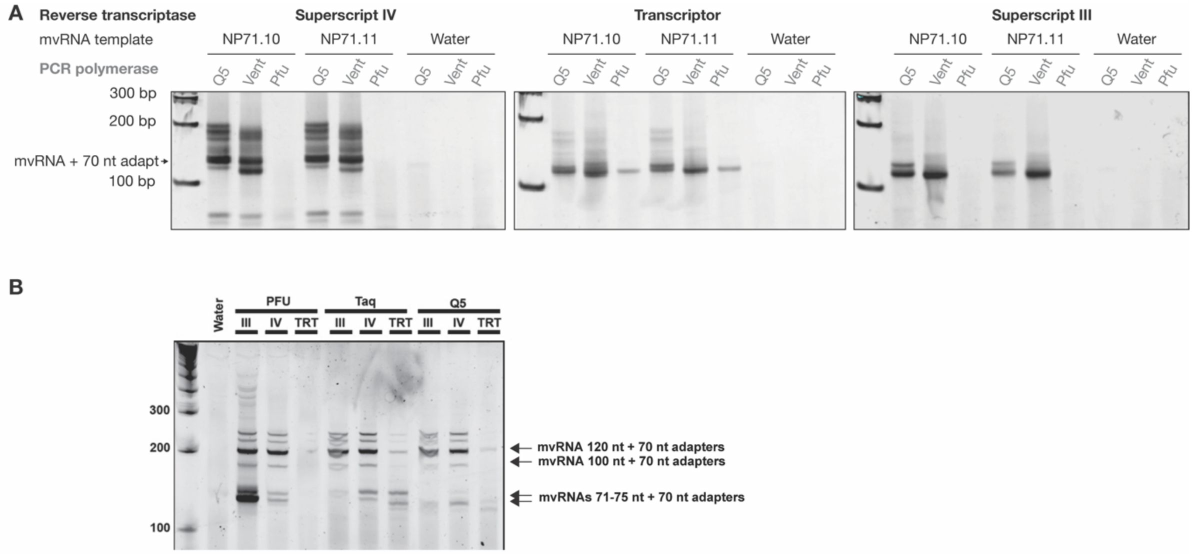

Figure S1. Detection of synthetic mvRNAs by different RT and PCR enzymes. A) Two synthetic mvRNAs were reverse transcribed into cDNA by three different RT enzymes and subsequently amplified by three different PCR enzymes. For sequences used, see Table S1. **B**) RT-PCR amplification of 4 different mvRNAs, with lengths 71, 75, 100 and 120 nt, by three different RT and PCR enzymes. Expected sizes and the length of PCR primers are indicated. RT enzymes used were superscript III (III), superscript IV (IV), Transcriptor (TRT). PCR enzymes uses were Pfu polymerase (PFU), Taq polymerase (Taq), and Q5 polymerase (Q5).

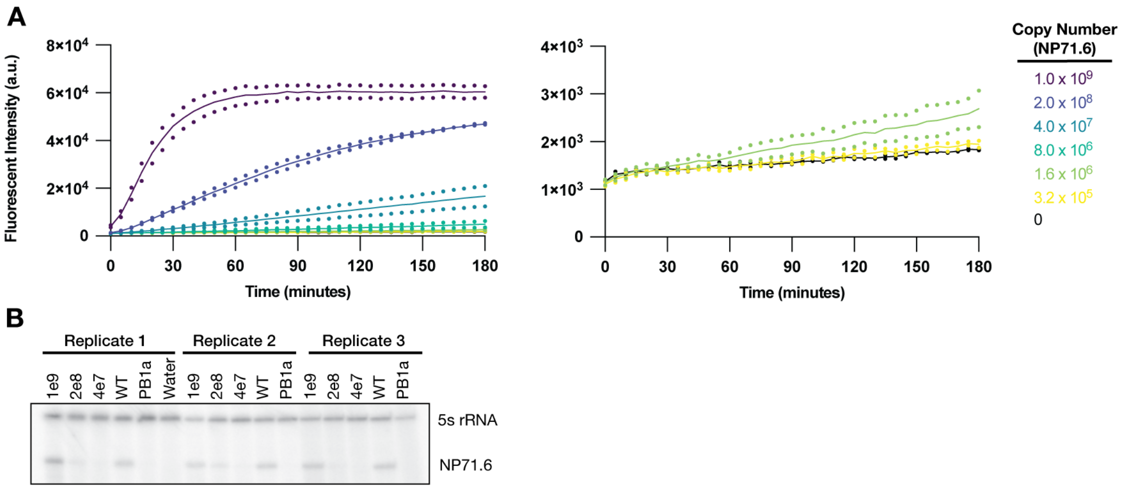

Figure S2. A) LbuCas13a detection of synthetic mvRNA NP71.6 diluted in HEK293T total RNA. Right graph is a close-up of bottom part of left graph. **B**) Detection of mvRNA NP71.6 by primer extension following plasmid-based expression in HEK293T cells in the presence of WT or an inactive (PB1a) WSN RNA polymerase. 5S rRNA was used as loading control.

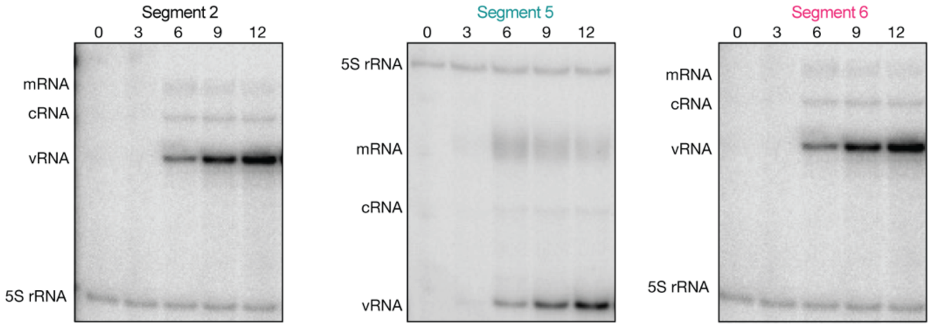

Figure S3. Replication and transcription products produced during WSN infection of A549 cells as determined by primer extension and denaturing PAGE. Time point post infection are indicated above each gel image.

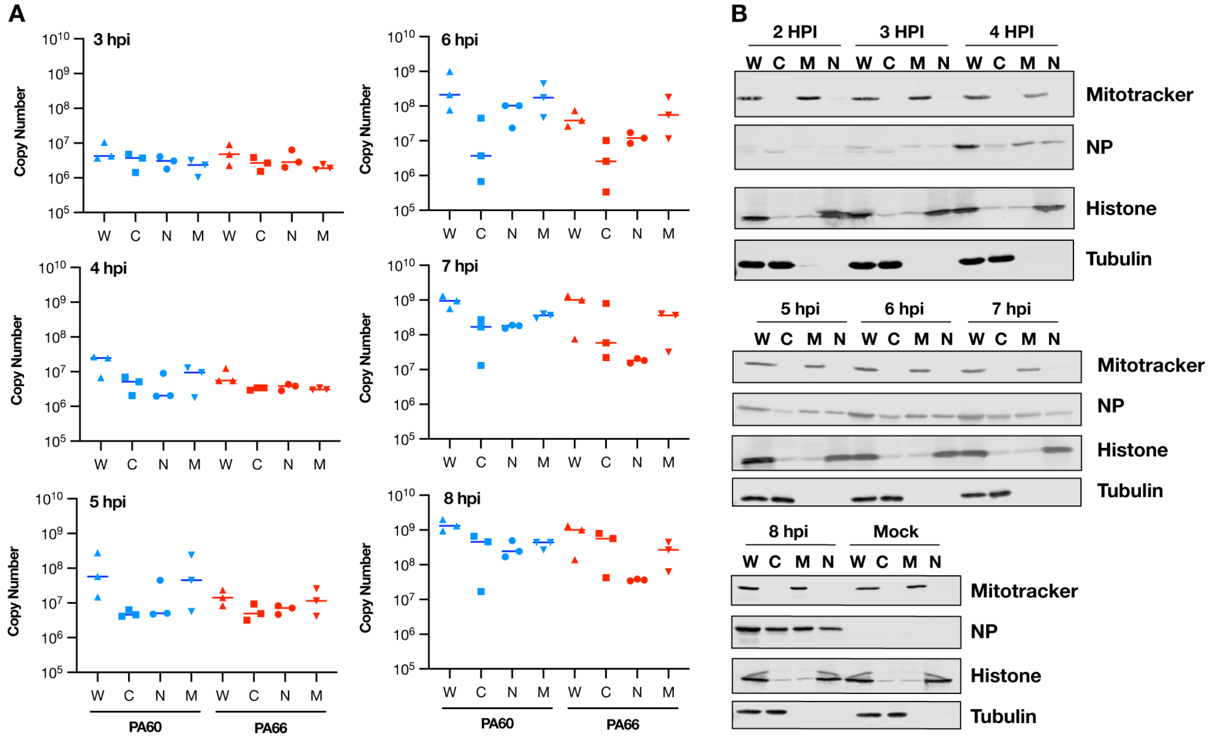

Figure S4. Detection of mvRNAs in influenza A virus infection of A549 cells using LbuCas13a. **A**) Cas13 fluorescent signals were converted from copy number per 75 ng of input RNA to the copy number per ul extracted sample to facilitate a direct comparison between the subcellular fractions. **B**) Western blot analysis of WSN infected A549 cell fractionation. Fractions are abbreviated as: W = whole cell, C = cytoplasmic, M = mitochontrial, N = nuclear.

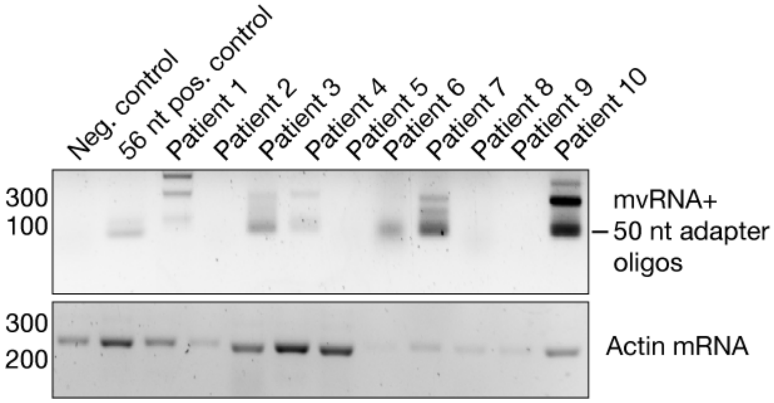

Figure S5. Detection of mvRNAs in influenza A virus positive clinical samples using RT-PCR. A 56 nt long segment 5-based mvRNA was used as positive control. As negative control, a clinical sample was used that had tested negative for influenza A virus genetic material.

Supplementary Tables

Table S1. Sequences of mvRNAs studied.

Name	Lab Reference	Sequence (5′ to 3′)	Figure
NP71.10	GC50_13	AGUAGAAACAAGGGUAUUUUUCUUUACUAGUGGCAGCAAAAGCAGGGUAACUAGUCUACCCUGCUUUUGCU	S1A
NP71.11	GC50_15	AGUAGAAACAAGGGUAUUUUUCUUUACUAGUGGCAGCAAAAGCACCCAUACUAGUCUACCCUGCUUUUGCU	S1A
NP71.6	GC50.9	AGUAGAAACAAGGGUAUUUUUCUUUACUAGUCCGGCCGUUUUGGUUGCCACUAGUCUACCCUGCUUUUGCU	2B-2F, S2
NP61	vNP61	AGUAGAAACAAGGGUAUUUUUCGAUGUCACUCUGUGAGUGAUUAUCUACCCUGCUUUUGCU	2G, 5A,5C-D, 6
PA-60	PA60	AGUAGAAACAAGGUACUUUUUUGGACAGUAUGCCAUUUUGAAUCAGUACCUGCUUUCGCU	3A-C, 4, S4, 5B
PA-66	PA66	AGUAGAAACAAGGUACUUUUUUGGACAGUAUGGAUAGCACAUUUUGAAUCAGUACCUGCUUUCGCU	3A-C,4, S4, 5B
PB1-64	PB1-64	AGUAGAAACAAGGCAUUUUUUCAGUCGGAUUGACAUCCAUUCAAAUGGUUUGCCUGCUUUCGCU	3A-B, D
PB1-66	PB1-66	AGUAGAAACAAGGCAUUUUUUCAUGAAGGACAAGCUAAACAUUCAAAUGGUUUGCCUGCUUUCGCU	3A, B, D
HA-61	vHA61	AGUAGAAACAAGGGUGUUUUUUAGAUCCAUUAUGUCUUUGUCACCCUGCUUUUGCU	3A, B, E
HA-64	vHA64	AGUAGAAACAAGGGUGUUUUUUAUCUGAAAGCUUGACACAGUGUUUGGAUCCAUUAUGUCUUUGUCACCCUGCUUUUGCU	3A, B, E
NS-56	vNS56	AGUAGAAACAAGGGUGUUUUUCCUUAUAUUUCUGAAAUCCUAAUCUUCCCCUGCUUUUGCU	3A, B, F
NS-80	vNS80	AGUAGAAACAAGGGUGUUUUUCCUUAUAUUUCUGAAAUCCUAAUCUCAUUCCCCUGCUUUUGCU	3A, B, F
mvRNA75	mvRNA75	AGTAGAAACAAGGGCACTACTGGAAAACTACCTGTTCCATGGCCAACACTTGTCACTACTTTCCCTGCTTTTGCT	S1B

mvRNA100	mvRNA100	AGTAGAAACAAGGGTAACAGCTGCTGGGATTACACATGGCATGGATGAACTATACAAATAAATGTCCAGACCTGCAGGCATGCAAGCTCCTGCTTTTGCT	S1B
mvRNA120	mvRNA120	AGTAGAAACAAGGGCGATGGCCCTGTCCTTTTACCAGACAACCATTACCTGTCCACACAATCTGCCCTTTCGAAAGATCCCAACGAAAAGAGAGACCACATGGTCCTTCCTGCTTTTGCT	S1B

Table S2. crRNA sequences used.

Name	Target	Sequence (5′ to 3′)	Limit of Detection (copies) (lowest standard that was 3 standard deviations above mock)	Figure
CL06	Human 5s rRNA*	GAUUUAGACUACCCCAAAAACGAAGGGGACUAAAACGGGCGCGUUCAGGGUGGUAUGGCCGUAG	Not determined	2A
CL11	Human 5s rRNA	GACCACCCCAAAAAUGAAGGGGACUAAAACGGGCGCGUUCAGGGUGGUAUGGCCGUAG	Not determined	2A
CL09	NP71.6	GACCACCCCAAAAAUGAAGGGGACUAAAACUAGACUAGUGGCAACCAAAACGGCCGGA	3e5	2B-F, S2A
CL51	NP-61	GACCACCCCAAAAAUGAAGGGGACUAAAACAGAUAAUCACUCACAGAGUGACAUCGAA	6e4	2G, 5A, C, D, 6
CL12	PA-60	GACCACCCCAAAAAUGAAGGGGACUAAAACGGUACUGAUUCAAAAUGGCAUACUGUCC	3e5	3A-C,4, S4, 5A
CL13	PA-66	GACCACCCCAAAAAUGAAGGGGACUAAAACGGUACUGAUUCAAAAUGUGCUAUCCAUA	3e5	3A-C,4, S4, 5A
CL14	PB1-64	GACCACCCCAAAAAUGAAGGGGACUAAAACAAUGGAUG	3e5	3A, B, D

		UCAAUCCGACUGAAAAAAU<br>G		
CL15	PB1-66	GACCACCCCAAAAAUGAAG<br>GGGACUAAAACAUUUGAAU<br>GUUUAGCUUGUCCUUCAUG<br>A	3e5	3A,<br>B, D
CL25	HA-61	GACCACCCCAAAAAUGAAG<br>GGGACUAAAACAAAGCAGG<br>GGAAGAUUAGGAUUUCAGA<br>A	3e5	3A,<br>B, E
CL26	HA-64	GACCACCCCAAAAAUGAAG<br>GGGACUAAAACAAAGCAGG<br>GGAAUGAGAUUAGGAUUUC<br>A	3e5	3A,<br>B, E
CL23	NS-56	GACCACCCCAAAAAUGAAG<br>GGGACUAAAACACAAAGAC<br>AUAAUGGAUCUAAAAAACAC	3e5	3A,<br>B, F
CL24	NS-80	GACCACCCCAAAAAUGAAG<br>GGGACUAAAACACUGUGUC<br>AAGCUUUCAGAUAAAAAACA	8e6	3A,<br>B, F

[*]LwaCas13a enzyme rather than LbuCas13a enzyme used for the other crRNAs.

Table S3. Other oligonucleotides used.

**Name**	**Description**	**Sequence (5′ to 3′)**	**Figure**
6UFAM	RNA reporter	/FAM/UUUUUU/3IABKFQ/	2, 3, 4, 5, S2, S4
GC-	Targets NP segment	AGCAAAAGCAGGGTAGACTAGT	2D, 2F, S2B
NP 5′	Targets NP segment	AGTAGAAACAAGGGTATTTTTC	2D, 2F, S2B
GC50.9 probe	Targets NP71.6	/FAM/TTACTAGTC/ZEN/CGGCCGTTTTGGTTGC/3IABKFQ/	2D-F
Lv3ga	vRNA RT primer (contains LNA)	GTTCAGACGTGTGCTCTTCCGATCTAGCG+AAAGCAGG	1A, S1, S5
Lv3aa	vRNA RT primer (contains LNA)	GTTCAGACGTGTGCTCTTCCGATCTAGC+A+AAAGCAGG	1A, S1, S5
Lv5	vRNA forward primer (contains LNA)	CACGACGCTCTTCCGATCTHNNNNNNNAGTAGAA+A+CAAGG	1A, S1, S5
P5short	vDNA fw	GAGATCTACACTCTTTCCCTACACGACGCTCTTCCGATCT	1A, S1, S5
I7short	vDNA rv	GAGATACTGGTGTGACTGGAGTTCAGACGTGTGCTCTTCCGATCT	1A, S1, S5

Table S4. Results of influenza patient clinical sample analysis.

Fig. Code	Internal Code	Cambridge Code	H1 or H3	Age (years)	Clinical Ct Value	Sex	Total RNA input (ng)	Copies/ uL
**H3-1**	C1	1193	H3	7	11	F	83.8	3521682.513
**H3-2**	C2	1392	H3	62	15	F	31.5	Not Detected
**H1-1**	C3	1109	H1	18	12	F	64.5	6162069.391
**H1-2**	C4	1275	H1	28	16	M	39.1	954004.4947
**H3-3**	C5	1196	H3	83	17	F	29.7	2238521.406
**H1-3**	C6	1163	H1	1	17	M	143.2	786978.3698
**H1-4**	C7	1268	H1	1	16	M	131.2	1182889.721
**H3-4**	C8	1375	H3	76	13	M	52.3	718879.6206
**H3-5**	C9	1497	H3	18	12	F	241	763487.979
**H1-5**	C10	1127	H1	76	17	M	86.7	2406890.514
**H3-6**	C11	1134	H3	88	14	F	45.8	674301.8301
**H1-6**	C12	1135	H1	57	17	F	31.9	838687.1017
**H3-7**	C13	1217	H3	71	10	F	24.4	4498742.275
**H3-8**	C14	1169	H3	41(days)	10	M	45.3	1279877.109
**H1-7**	C15	1175	H1	15	17	M	35.3	857500.4898
**H1-8**	C16	1139	H1	35	16	F	113.1	8142113.409
**H1-9**	C17	1248	H1	58	16	F	56	737658.3474
**H1-10**	C18	1219	H1	79	17	M	43.2	794027.1421
**H3-9**	C19	1229	H3	2	13	M	118.1	2219306.633
**H1-11**	C20	1448	H1	42	13	F	153.1	3399464.279
**H1-12**	C21	1126	H1	84	29	F	67.0	Not detected
**H3-10**	C22	1588	H3	82	10	F	39.9	4215913.226
**H1-13**	C23	1464	H1	87	14	F	80.6	2535752.682
**H1-14**	C24	1403	H1	34	15	F	74.1	545581.3123
**H3-11**	C25	1608	H3	87	12	F	148.0	564835.1674
**H1-15**	C26	1633	H1	29	15	F	75.0	4014859.47
**H3-12**	C27	1673	H3	32	13	F	173.1	531682.0197
**H1-16**	C28	1314	H1	23	17	F	162.2	640879.3779
**H1-17**	C29	1435	H1	3	16	F	45.2	6224905.957
**H1-18**	C30	1689	H1	81	15	F	80.3	1378330.248
