# Supplementary material for: Cas13-based amplification-free quantification of aberrant RNAs during influenza virus infection
Source: bioRxiv. 2023 Nov 3:2023.11.03.565460. Preprint. [Version 1] doi: 10.1101/2023.11.03.565460 (PMC10635118; doi:10.1101/2023.11.03.565460)
Supplement: Supplementary file 1 [file media-1.pdf]

Supplemental information for:

Cas13-based amplification-free quantification of aberrant RNAs during influenza virus infection

Caitlin H. Lamb[1], Emmanuelle M. Pitré[1,2], Elizaveta Elshina[1], Charlotte V. Rigby[1,2,3], Karishma Bisht[1], Hamid Jalal[3], Cameron Myhrvold[1,4,5,6,*], Aartjan J.W. te Velthuis[1,*]

[1] Department of Molecular Biology, Princeton University, Princeton, NJ 08544

[2] University of Cambridge, Department of Pathology, Addenbrooke's Hospital, Cambridge CB2 2QQ, United Kingdom

[3] Public Health England, Addenbrooke's Hospital, Cambridge CB2 2QQ, United Kingdom

[4] Department of Chemical and Biological Engineering, Princeton University, Princeton, NJ 08544

[5] Omenn-Darling Bioengineering Institute, Princeton University, Princeton, NJ 08544

[6] Department of Chemistry, Princeton University, Princeton, NJ 08544

[*]Address correspondence to: A.J.W.t.V. (aj.te.velthuis@princeton.edu); C.M. (cmyhrvol@princeton.edu)

Supplementary Figures

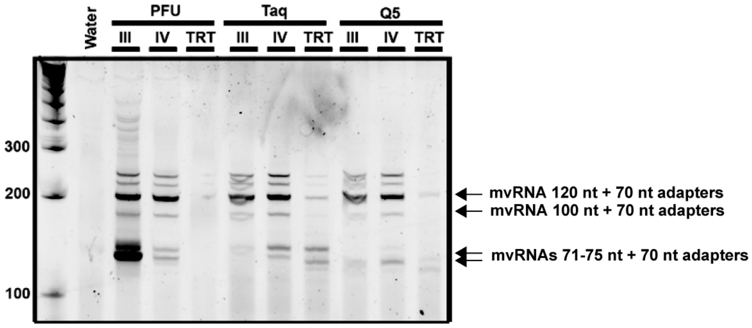

Figure S1. RT-PCR amplification of 4 different mvRNAs, with lengths 71, 75, 100 and 120 nt, by three different RT and PCR enzymes. Expected sizes and the length of PCR primers are indicated. RT enzymes used were superscript III (III), superscript IV (IV), Transcriptor (TRT). PCR enzymes uses were Pfu polymerase (PFU), Taq polymerase (Taq), and Q5 polymerase (Q5).

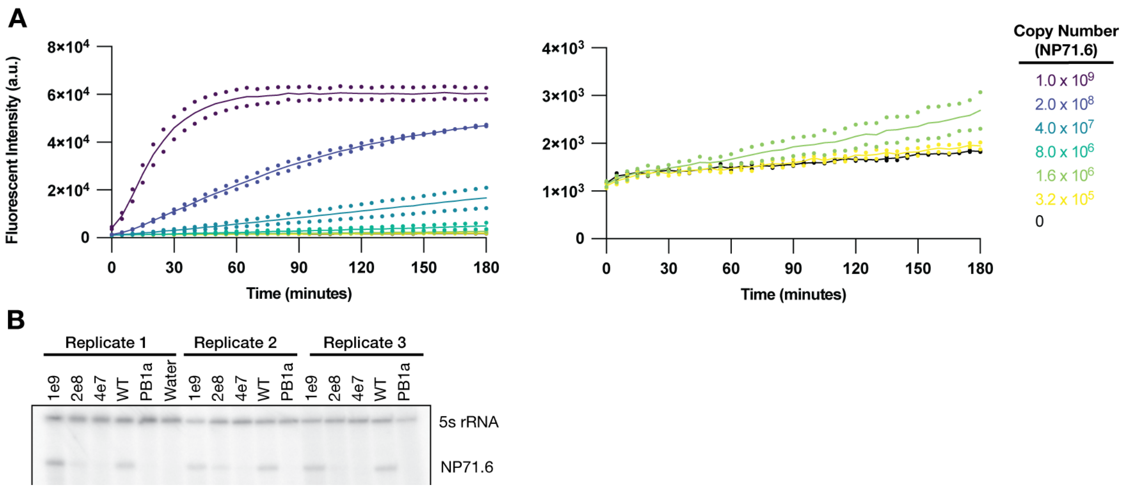

Figure S2. A) LbuCas13a detection of synthetic mvRNA NP71.6 diluted in HEK293T total RNA. Right graph is a close-up of bottom part of left graph. **B**) Detection of mvRNA NP71.6 by primer extension following plasmid-based expression in HEK293T cells in the presence of WT or an inactive (PB1a) WSN RNA polymerase. 5S rRNA was used as loading control.

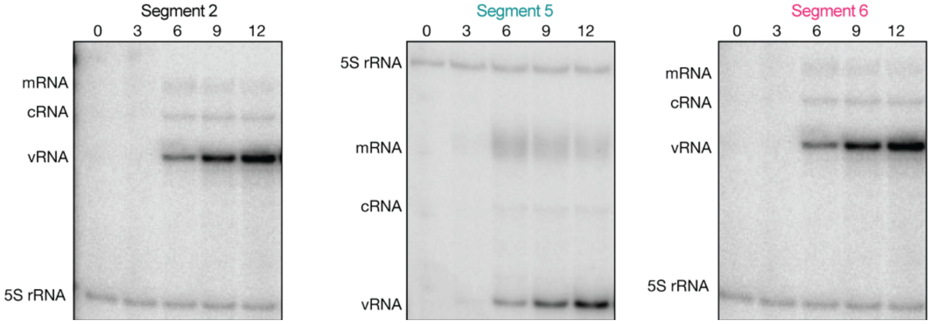

Figure S3. Replication and transcription products produced during WSN infection of A549 cells as determined by primer extension and denaturing PAGE. Time point post infection are indicated.

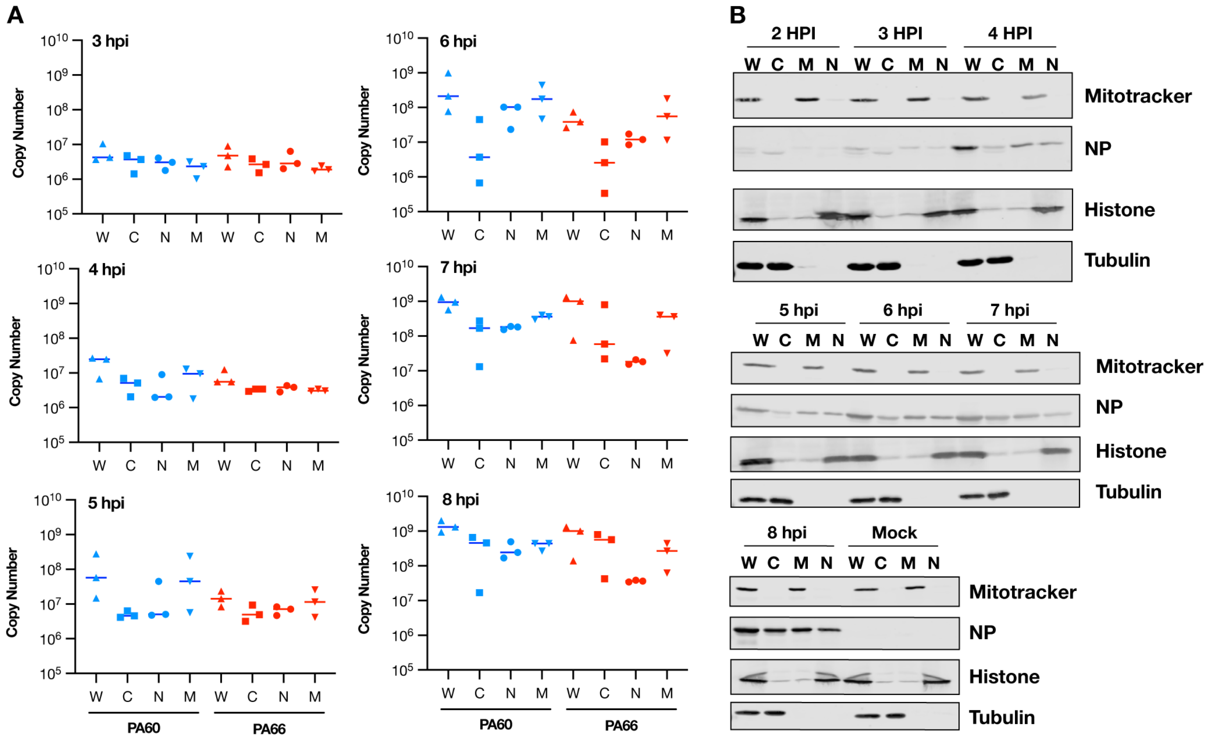

Figure S4. Detection of mvRNAs in influenza A virus infection of A549 cells using LbuCas13a. Signals were converted from copy number per 75 ng of input RNA to the copy number per ul extracted sample to facilitate a direct comparison between the subcellular fractions. B) Western blot analysis of WSN infected A549 cell fractionation.

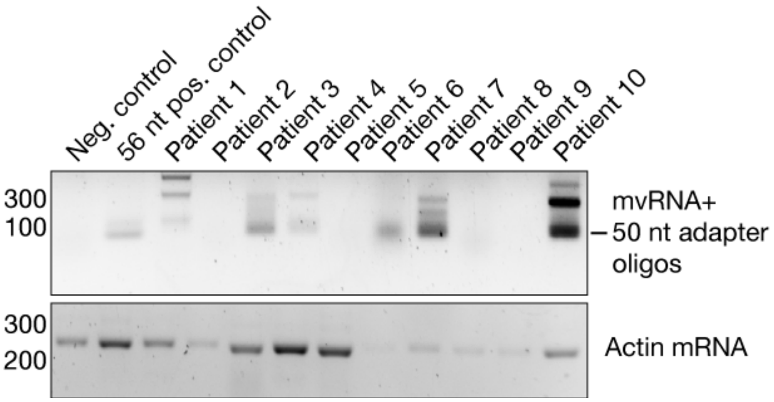

Figure S5. Detection of mvRNAs in influenza A virus positive clinical samples using RT-PCR. A 56 nt long segment 5-based mvRNA was used as positive control. As negative control, a clinical sample was used that had tested negative for influenza A virus genetic material.

Supplementary Tables

Table S1. Sequences of mvRNAs studied.

Name	Lab Reference	Sequence (5′ to 3′)	Figure
NP71.10	GC50_13	AGUAGAAACAAGGGUAUUUUUCUUUACUAGUGGCAGCAAAAGCAGGGUAACUAGUCUACCCUGCUUUUGCU	1A
NP71.11	GC50_15	AGUAGAAACAAGGGUAUUUUUCUUUACUAGUGGCAGCAAAAGCACCCAUACUAGUCUACCCUGCUUUUGCU	1A
NP71.6	GC50.9	AGUAGAAACAAGGGUAUUUUUCUUUACUAGUCCGGCCGUUUUGGUUGCCACUAGUCUACCCUGCUUUUGCU	2B-2F
NP61	vNP61	AGUAGAAACAAGGGUAUUUUUCGAUGUCACUCUGUGAGUGAUUAUCUACCCUGCUUUUGCU	2G, 5B-G
PA-60	PA60	AGUAGAAACAAGGUACUUUUUUGGACAGUAUGCCAUUUUGAAUCAGUACCUGCUUUCGCU	3A-C, 4, 5A, S4
PA-66	PA66	AGUAGAAACAAGGUACUUUUUUGGACAGUAUGGAUAGCACAUUUUGAAUCAGUACCUGCUUUCGCU	3A-C,4, S4, 5A
PB1-64	PB1-64	AGUAGAAACAAGGCAUUUUUUCAGUCGGAUUGACAUCCAUUCAAAUGGUUUGCCUGCUUUCGCU	3A-B, D
PB1-66	PB1-66	AGUAGAAACAAGGCAUUUUUUCAUGAAGGACAAGCUAAACAUUCAAAUGGUUUGCCUGCUUUCGCU	3A-B, D
HA-61	vHA61	AGUAGAAACAAGGGUGUUUUUUAGAUCCAUUAUGUCUUUGUCACCCUGCUUUUGCU	3A, B, E
HA-64	vHA64	AGUAGAAACAAGGGUGUUUUUUAUCUGAAAGCUUGACACAGUGUUUGGAUCCAUUAUGUCUUUGUCACCCUGCUUUUGCU	3A, B, E
NS-56	vNS56	AGUAGAAACAAGGGUGUUUUUCCUUAUAUUUCUGAAAUCCUAAUCUUCCCCUGCUUUUGCU	3A-B, 3F
NS-80	vNS80	AGUAGAAACAAGGGUGUUUUUCCUUAUAUUUCUGAAAUCCUAAUCUCAUUCCCCUGCUUUUGCU	3A-B, 3F
mvRNA75	mvRNA75	AGTAGAAACAAGGGCACTACTGGAAAACTACCTGTTCCATGGCCAACACTTGTCACTACTTTCCCTGCTTTTGCT	S1
mvRNA100	mvRNA100	AGTAGAAACAAGGGTAACAGCTGCTGGGATTACACATGGCATGGATGAACTATACAAATAAATGTCCAGACCTGCAGGCATGCAAGCTCCTGCTTTTGCT	S1
mvRNA120	mvRNA120	AGTAGAAACAAGGGCGATGGCCCTGTCCTTTTACCAGACAACCATTACCTGTCCACACAATCTGCCCTTTCGAAAGATCCCAACGAAAAGAGAGACCACATGGTCCTTCCTGCTTTTGCT	S1

Table S2. crRNA sequences used.

Name	Target	Sequence (5′ to 3′)	Figure
CL06	Human 5s rRNA*	GAUUUAGACUACCCCAAAAACGAAGGGGACUAAAAC GGGCGCGUUCAGGGUGGUAUGGCCGUAG	2A
CL11	Human 5s rRNA	GACCACCCCAAAAAUGAAGGGGACUAAAACGGGCG CGUUCAGGGUGGUAUGGCCGUAG	2A
CL09	NP71.6	GACCACCCCAAAAAUGAAGGGGACUAAAACUAGACU AGUGGCAACCAAAACGGCCGGA	2B-F
CL51	NP-61	GACCACCCCAAAAAUGAAGGGGACUAAAACAGAUAA UCACUCACAGAGUGACAUCGAA	2G, 5B-G
CL12	PA-60	GACCACCCCAAAAAUGAAGGGGACUAAAACGGUAC UGAUUCAAAAUGGCAUACUGUCC	3A-C,4, S4, 5A
CL13	PA-66	GACCACCCCAAAAAUGAAGGGGACUAAAACGGUAC UGAUUCAAAAUGUGCUAUCCAUA	3A-C,4, S4, 5A
CL14	PB1-64	GACCACCCCAAAAAUGAAGGGGACUAAAACAAUGGA UGUCAAUCCGACUGAAAAAAUG	3A-B, 3D
CL15	PB1-66	GACCACCCCAAAAAUGAAGGGGACUAAAACAUUUGA AUGUUUAGCUUGUCCUUCAUGA	3A-B, 3D
CL25	HA-61	GACCACCCCAAAAAUGAAGGGGACUAAAACAAAGCA GGGGAAGAUUAGGAUUUCAGAA	3A-B, 3E
CL26	HA-64	GACCACCCCAAAAAUGAAGGGGACUAAAACAAAGCA GGGGAAUGAGAUUAGGAUUUCA	3A-B, 3E
CL23	NS-56	GACCACCCCAAAAAUGAAGGGGACUAAAACACAAAG ACAUAAUGGAUCUAAAAAACAC	3A-B, 3F
CL24	NS-80	GACCACCCCAAAAAUGAAGGGGACUAAAACACUGU GUCAAGCUUUCAGAUAAAAAACA	3A-B, 3F

*LwaCas13a enzyme rather than LbuCas13a enzyme used for the other crRNAs.

Table S3. Other oligonucleotides used.

Name	Description	Sequence (5′ to 3′)	Figure
6UFAM	RNA reporter	/FAM/UUUUUU/3IABKFQ/	2, 3, 4, 5, S2, S4
GC-	Targets NP segment	AGCAAAAGCAGGGTAGACTAGT	2D, 2F, S2B
NP 5′	Targets NP segment	AGTAGAAACAAGGGTATTTTTC	2D, 2F, S2B
GC50.9 probe	Targets NP71.6	/FAM/TTACTAGTC/ZEN/CGGCCGTTTTGGTTGC/3IABKFQ/	2D-F
Lv3ga	vRNA RT primer (contains LNA)	GTTCAGACGTGTGCTCTTCCGATCTAGCG+AAAGCAGG	1A, S1, S5
Lv3aa	vRNA RT primer (contains LNA)	GTTCAGACGTGTGCTCTTCCGATCTAGC+A+AAAGCAGG	1A, S1, S5
Lv5	vRNA forward primer (contains LNA)	CACGACGCTCTTCCGATCTHNNNNNNNAGTAGAA+A+CAAGG	1A, S1, S5
P5short	vDNA fw	GAGATCTACACTCTTTCCCTACACGACGCTCTTCCGATCT	1A, S1, S5
I7short	vDNA rv	GAGATACTGGTGTGACTGGAGTTCAGACGTGTGCTCTTCCGATCT	1A, S1, S5

Table S4. Results of clinical sample analysis.

Code	Internal Code	Cambridge Code	H1 or H3	Reported CT Value	Age (years)*	Sex	Total RNA input (ng)	Copies/µL
**H3-1**	C1	1193	H3	11	7	F	83.8	3521683
**H3-2**	C2	1392	H3	15	62	F	31.5	n/a
**H1-1**	C3	1109	H1	12	18	F	64.5	6162069
**H1-2**	C4	1275	H1	16	28	M	39.1	n/a
**H3-3**	C5	1196	H3	17	83	F	29.7	2238521
**H1-3**	C6	1163	H1	17	1	M	143.2	n/a
**H1-4**	C7	1268	H1	16	1	M	131.2	n/a
**H3-4**	C8	1375	H3	13	76	M	52.3	n/a
**H3-5**	C9	1497	H3	12	18	F	241	n/a
**H1-5**	C10	1127	H1	17	76	M	86.7	2406890
**H3-6**	C11	1134	H3	14	88	F	45.8	n/a
**H1-6**	C12	1135	H1	17	57	F	31.9	n/a
**H3-7**	C13	1217	H3	10	71	F	24.4	4498742
**H3-8**	C14	1169	H3	10	41(days)	M	45.3	1279877
**H1-7**	C15	1175	H1	17	15	M	35.3	n/a
**H1-8**	C16	1139	H1	16	35	F	113.1	8142113
**H1-9**	C17	1248	H1	16	58	F	56	n/a
**H1-10**	C18	1219	H1	17	79	M	43.2	n/a
**H3-9**	C19	1229	H3	13	2	M	118.1	2219306

*Unless otherwise noted
